# Anti-Cholera Vaccination

**Published:** 1915-08

**Authors:** 


**Anti-cholera Vaccination.** Dopter, Paris Medicale. In the Balkan wars, Armand reported the incidence of cholera among

14,332 unvaccinated officers and enlisted men as 5.75 per cent., among 21,216 men vaccinated once 3.12, and among 72,652 vaccinated twice 0.43. In the civil population of Greece, Cardamatis reported analogous percentages of 2.12, 0.26, and 0.01 respectively. The vaccine used in these cases was prepared at the Pasteur Institute and consisted of cultures on agar heated to 60° C., for an hour. Dopter says three injections at intervals of five days are essential to establish immunity. Doses of 1, 1.5, and 2 cubic centimetres respectively, should be administered in succession.

